# A Super Stable Mutant of the Plant Protein Monellin Endowed with Enhanced Sweetness

**DOI:** 10.3390/life11030236

**Published:** 2021-03-12

**Authors:** Masoud Delfi, Alessandro Emendato, Serena Leone, Eros Antonio Lampitella, Piero Porcaro, Gaetano Cardinale, Luigi Petraccone, Delia Picone

**Affiliations:** 1 Department of Chemical Sciences, University of Naples “Federico II”, Complesso Universitario Monte S. Angelo, Via Cintia, 80126 Napoli, Italy; masoud.delfi@unina.it (M.D.); alessandro.emendato@unina.it (A.E.); serena.leone@szn.it (S.L.); erosantoniolampitella@yahoo.it (E.A.L.); luigi.petraccone@unina.it (L.P.); 2 Consorzio Sannio Tech, Strada Statale Appia Km 256 n.7, Apollosa, 82010 Benevento, Italy; piero.porcaro@tecnobios.com (P.P.); gaetano.cardinale@tecnobios.com (G.C.)

**Keywords:** sweet proteins, single-chain monellin (MNEI), sensory analysis, shelf life, high intensity sweeteners, thermochemical stability

## Abstract

Sweet proteins are a class of proteins with the ability to elicit a sweet sensation in humans upon interaction with sweet taste receptor T1R2/T1R3. Single-chain Monellin, MNEI, is among the sweetest proteins known and it could replace sugar in many food and beverage recipes. Nonetheless, its use is limited by low stability and high aggregation propensity at neutral pH. To solve this inconvenience, we designed a new construct of MNEI, dubbed Mut9, which led to gains in both sweetness and stability. Mut9 showed an extraordinary stability in acidic and neutral environments, where we observed a melting temperature over 20 °C higher than that of MNEI. In addition, Mut9 resulted twice as sweet than MNEI. Both proteins were extensively characterized by biophysical and sensory analyses. Notably, Mut9 preserved its structure and function even after 10 min boiling, with the greatest differences being observed at pH 6.8, where it remained folded and sweet, whereas MNEI lost its structure and function. Finally, we performed a 6-month shelf-life assessment, and the data confirmed the greater stability of the new construct in a wide range of conditions. These data prove that Mut9 has an even greater potential for food and beverage applications than MNEI.

## 1. Introduction

It is commonly believed that sweetness can be perceived only upon consumption of sugars or non-caloric sweeteners. However, some plant proteins are actually sweeter than sucrose and most non-caloric sweeteners by orders of magnitude [1]. To date, four sweet proteins have been isolated and purified from unrelated tropical plants: monellin [2], thaumatin [3], brazzein [4] and mabinlin [5]. In addition, two sweet taste-modifying proteins, which upon ingestion can turn the flavor of sour substances into sweet, are also known: miraculin, and curculin [6,7,8,9,10]. Other than their plant origin, none of these proteins share any sequence or structure homology [11]; nonetheless, sweet and sweet taste-modifying proteins are able to elicit a sweet sensation upon binding the same sweet taste receptor, T1R2/T1R3, a heterodimeric G-protein coupled receptor located on specialized cells of the tongue, palate, pharynx, and gut [12,13,14,15]. Sugar and small sweeteners also activate the same receptor, but the binding modes of low molecular weight compounds and proteins are very different [16,17,18,19]. In fact, while small sweeteners bind to the orthosteric site of the receptor, sweet proteins, with their considerably larger dimensions, are believed to allosterically activate it. The model that best describes this mode of action is known as the “wedge model” [17,20,21], according to which the sweet taste receptor is in equilibrium between an active and an inactive conformation and sweet proteins stabilize the active conformation by binding a wide cleft bridging both subunits of the receptor. Although never experimentally proven, the wedge model has so far allowed for the prediction of several sweeter mutants of monellin, thaumatin and brazzein [22,23,24].

Recently, the prevalence of some hazardous diet-related diseases, such as obesity, diabetes, hyperlipidemia and caries, has pushed scientists and food and beverage companies to seek healthier replacements for sugar and ordinary sweeteners. This is why natural sweeteners, like sweet proteins, have attracted much attention, especially since recent studies have pointed out that traditional artificial sweeteners may be related to adverse health effects [25,26]. On the other hand, natural sweet proteins also present many disadvantages that have hampered their use in large scale processes, especially concerning their limited availability and scarce resistance to factors like temperature and pH. Deep structural studies of receptor–protein complexes in parallel with protein engineering techniques are the key for building new, enhanced constructs that could find wide use in food and beverage products. According to the wedge model, these constructs should at all times preserve the 3D shape of the original proteins to retain their functionality. In addition, surface charge compatibility with the T1R2/T1R3 dimer should always be minded, since this is the key to the allosteric activation of the receptor [17,21]. For instance, mutations increasing the acidic character of monellin [27,28,29,30], thaumatin [23,31,32,33], and brazzein [24,34,35] often lead to a decrease or cancellation of sweetness, since the binding surface on the receptor presents several acidic patches. On the other hand, since the distribution of acidic amino acids on the surface of sweet taste receptor is uneven, the random introduction of basic amino acids on the external surface of sweet proteins might also abate sweetness [27].

We focused our attention on one of the sweetest and best characterized proteins known to date, monellin, which is about 100,000 times sweeter than sucrose on a molar basis [11]. The native protein is extracted and purified from *Dioscoreophyllum cumminsii*, also known as serendipity berry, a plant from tropical rainforests [36]. It is a small (~11 kDa) globular protein composed of two polypeptide chains, A and B, of 45 and 50 amino acid residues, respectively, linked together by non-covalent interactions. The 3D structure of monellin is characterized by a five-strand β-sheet half-wrapped around an α-helix [37,38,39]. This potent natural sweetener undergoes irreversible denaturation and loss of sweetness when heated above 50 °C [40]. To resolve this inconvenience, MNEI was designed by joining both subunits of the protein through a Gly Phe dipeptide linker to enhance its thermal stability [41]. Indeed, MNEI has a melting temperature of over 70 °C and, in certain conditions, can be heated without losing its sweetness [40]. Thanks to its distinct features, MNEI could be better than monellin as a substitute for commonly used sweeteners in industrial applications [42].

In this study, we aimed at further improving MNEI features, in particular thermal and chemical stability and sweetness, by applying targeted point mutations. Over the years, many research groups have performed extensive studies on MNEI and the effect of many point mutations has been examined, sometimes with stronger focus on stability gains, other times on flavor improvement [22,28,29,30,43,44]. Nonetheless, the results of these studies have rarely been compared and combined, and the additivity of the mutations producing gains in function has never been assessed. We reviewed the most promising results obtained with MNEI mutants and carefully selected some of the best performing constructs in order to combine them, yielding a “super mutant”. The mutations E23A, C41A, Y65R, and S76Y were selected so that they could be as widely and homogeneously spread on the protein surface as possible, thus producing the maximum gain in function while not interfering with each other and not affecting the overall structure. The new protein obtained, named Mut9, was expressed and characterized by different biophysical techniques, with particular attention to its thermal/chemical stability and sweetness potency. The results confirmed that Mut9 further improves the properties of MNEI, retaining most of the beneficial features previously reported for the individual point mutations. This moves us even closer to obtaining a protein sweetener with features that could comply with industry processes, in response to the growing demand for new sugar substitutes in food and beverage products.

## 2. Materials and Methods

### 2.1. Cloning, Expression and Purification of the Mutant

The synthetic full-length gene encoding for the sequence of Mut9 was purchased from Eurofins Genomics. The gene was cloned into the expression vector pET22b(+) (Novagen) between the *NdeI* and *BamHI* restriction sites. The recombinant protein was expressed in *Escherichia coli* BL21(DE3) and purified from the cell lysate by ion-exchange chromatography followed by size-exclusion chromatography for salt removal as previously described [45]. Protein identity and purity were confirmed by SDS-PAGE and circular dichroism spectroscopy. Protein concentration was measured using UV-Vis spectrophotometer (Thermo GENESYS^TM^ 10UV, Madison, WI, USA).

### 2.2. Circular Dichroism Spectroscopy (CD Spectroscopy)

CD measurements were performed on a Jasco J-715 spectropolarimeter (Jasco, Essex, UK), equipped with a Peltier temperature control system (PTC-348WI, Jasco, Essex, UK), using a 0.1 cm quartz cell. The CD curves of Mut9 were obtained in 0.020 M sodium phosphate buffer at pH 2.5, 5.1, and 6.8. To assess the effect of temperature, spectra of Mut9 and MNEI in 0.020 M sodium phosphate buffer at pH 2.5 and 6.8 were measured at 10 °C intervals in the range 25–95 °C and back to 25 °C. In another experiment, CD spectra were acquired upon boiling Mut9 and MNEI dissolved in the same buffers for 2, 5, and 10 min, and cooling back the protein solutions to room temperature. The spectra were taken in the far UV-range (195–250 nm) with a scan speed of 50 nm/min and each experiment was performed with 3 accumulations. Molar ellipticity per mean residue [θ] was calculated according to the formula:

[θ] = [θ]_obs_ mrw/(10 × l × C), deg cm^2^ dmol^−1^

where [θ]_obs_ is the raw ellipticity values measured in degrees, mrw is the mean residue molecular weight of each protein (Da), C is the protein concentration in g/mL and l is the optical path length of the quartz cell in cm. In all experiments, the concentration Mut9 and MNEI was 0.2 mg/mL, measured by UV absorbance at 280 nm using a value of the absorbance at 0.1% of 1.41. To have a quantitative estimation of the secondary structure content, the CD spectra were deconvoluted using an online tool [46].

### 2.3. Differential Scanning Calorimetry (DSC)

Calorimetric measurements were performed using a Nano-DSC 6300 (TA Instruments, New Castle, DE, USA). Protein samples were prepared in the appropriate buffer solutions with a concentration of 1 mg/mL and ran with a scanning speed of 1 °C/min and in a temperature range of 20–110 °C for Mut9 and 20–100 °C for MNEI. During the temperature scans a total pressure of 3.0 atm was applied to both cells using nitrogen gas. Buffer scans were recorded separately under the same conditions and subtracted from sample scans to obtain the excess molar heat capacity function [47]. A second run heating of the protein samples under identical conditions, after cooling down from the first run heating was also performed to verify the reversibility of the process.

The denaturation temperature, Tm and enthalpy Δ_d_H were obtained by the maximum of the DSC peak and the integrated area under the peak, respectively. All DSC data analysis were performed using the Nano-Analyze software supplied with the instrument.

### 2.4. Sensory Analysis

Sweetness intensity was evaluated by triangle test [27]. A team of five panelists participated in the sensory analysis. MNEI solutions and mineral water were used as positive and negative controls, respectively. Three paper cups, one containing 5 mL of protein sample and two cups containing 5 mL of mineral water were provided for the panelists to taste the samples and record their evaluation from 0 (no taste) to 5. A value of 1 indicated the perception of a taste, 2 meant the taste was recognized as sweet. The sample solutions were provided from the lowest (35 nM) to the highest (220 nM) concentration. Sweetness threshold was the concentration at which the protein scored 2 on average.

To assess taste performance upon thermal treatment, a blind sensory analysis was performed on 20 mg/L Mut9 and MNEI sample solutions before and after boiling for 2, 5, and 10 min. The boiled samples were tasted, and the assessments were made on a table with three tasting rates: same sweetness, decreased sweetness, and loss of sweetness. In both experiments, the subjects tested the sample solutions without any time constraints, then spat it out and rinsed their mouth thoroughly with mineral water within 1 min.

### 2.5. Shelf-Life Studies

The stability of Mut9 and MNEI was evaluated upon extended storage: samples of Mut9 and MNEI at the concentrations of 0.5 and 5.0 mg/mL were prepared at pH 2.5, 5.1, and 6.8 in 0.020 M phosphate buffers. The samples were stored for 6 months at 4 °C to simulate fridge storage, or at 37 °C for an accelerated shelf-life assessment. The protein concentration of the samples was measured using a UV-Vis spectrophotometer (Thermo GENESYS^TM^ 10UV, USA). Prior to each measurement, the samples were diluted 10 times by deionized water and the protein concentration was calculated using the UV absorbance at 280 nm. Protein content (%) = (measured protein concentration/Initial protein concentration) × 100.

## 3. Results

### 3.1. Protein Design and Production

**Figure 1:**
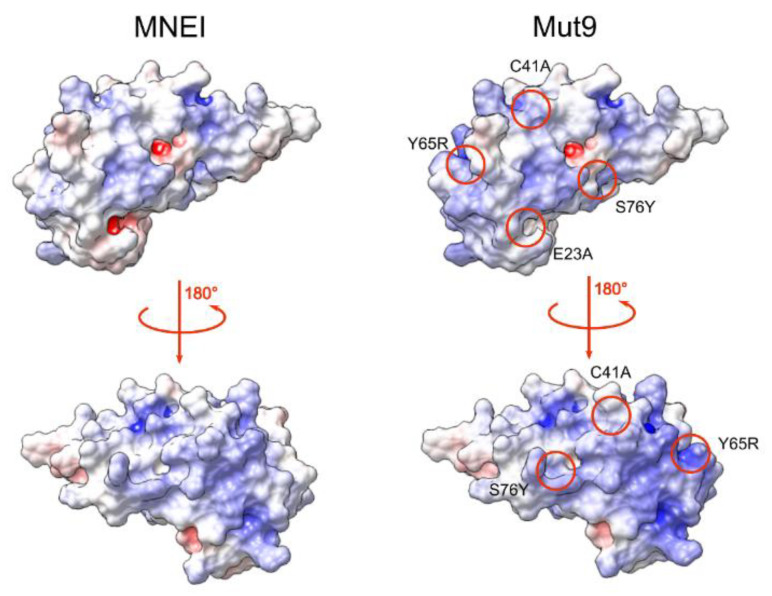
Position of the single point mutations in Mut9 (**right**) and their effect on the surface electrostatic potentials compared to MNEI (**left**). Electrostatic potential surfaces were calculated with APBS [49].

To design a new monellin derivative with higher sweetness and improved resistance to physical and chemical stressors, we first performed a careful review of the known mutants of MNEI. We selected four point mutations, namely E23A, C41A [48], Y65R [27], and S76Y [43], that were associated with significant gains in stability or taste. The construct containing the mutations was termed Mut9. The mutation sites were chosen so that they were as evenly distributed as possible on the protein surface, that they would not interact with each other, and they would not affect the global fold of MNEI. Figure 1 highlights the location of the amino acid substitutions in Mut9 and their effect on the electrostatic surface potential of MNEI.

Mut9 was expressed in *Escherichia coli* BL21 (DE3) using a standard recombinant expression protocol and purified by the same procedure already reported for the parent protein MNEI [50,51]. The only mentionable variation in the purification process is that Mut9 elutes from the cation exchange resin at a higher salt concentration (200 mM NaCl), according to the increased positive charge of the protein compared to MNEI, which elutes between 100 and 150 mM NaCl.

### 3.2. Secondary Structure Assessment

**Figure 2:**
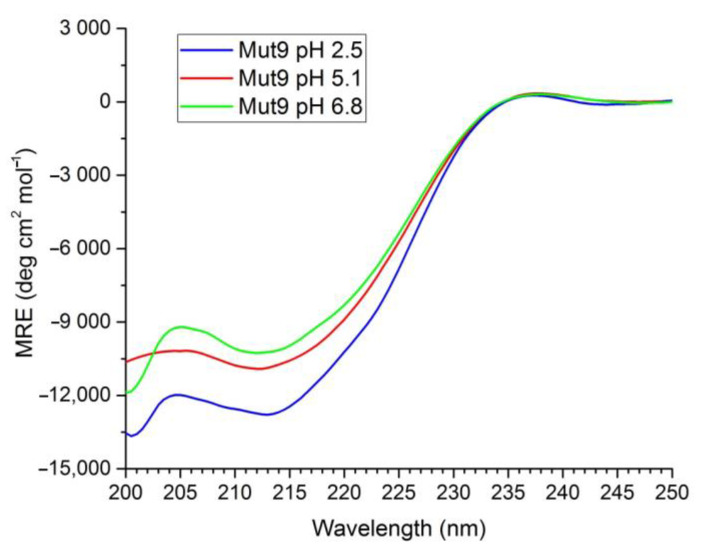
CD spectra of 0.2 mg/mL Mut9 solutions in 0.02 M sodium phosphate buffer at different pH values.

We assessed the folding and secondary structure content of Mut9 and MNEI from strongly acidic to almost neutral pH (i.e., 2.5, 5.1, and 6.8) by circular dichroism spectroscopy (CD). At all examined pHs, the spectra of Mut9 were characterized by two minima, located at 201 and 213 nm (Figure 2). To achieve quantitative estimations of the secondary structure contents of Mut9 at different pHs, the spectra were deconvoluted using the BestSel online tool [46]. The β-sheet and α-helix content showed minor changes from pH 2.5 to 6.8, confirming the stability of Mut9 fold in a wide pH range (Table 1).

### 3.3. Sensory Analysis

**Figure 3:**
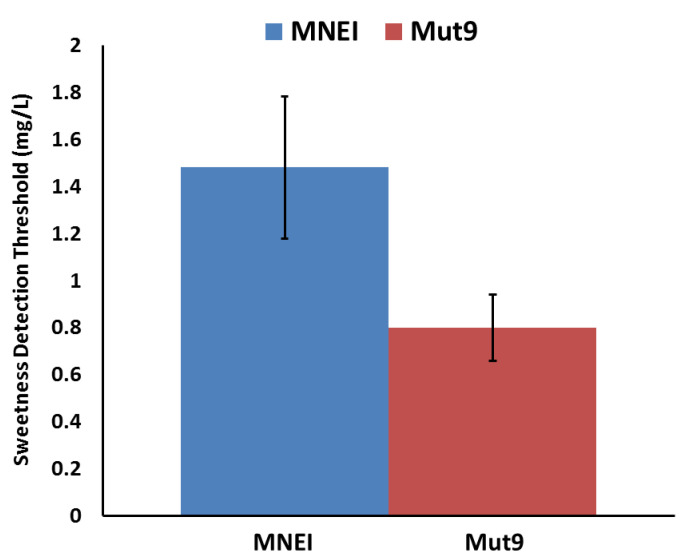
Sweetness detection thresholds of MNEI and Mut9.

To evaluate the effect of the mutations in terms of sweetness potency, Mut9 was subjected to a taste assessment. The sweetness threshold of Mut9 was evaluated by a panel of five tasters using the triangle test technique. According to the outcome, the sweetness threshold of Mut9 was 0.8 mg/L (71 nM). The same panel tasted MNEI as a positive control, and the sweetness threshold resulted in 1.48 mg/L (132 nM), which is in good agreement with literature data [52]. Based on these results, the point mutations included in Mut9 were able to increase the parent protein’s sweetness by almost 2 folds, in line with what observed for Y65R-MNEI. The presented column graph (Figure 3) shows the sweetness detection threshold of both proteins, MNEI and Mut9.

### 3.4. Thermal Stability Assessment

**Figure 4:**
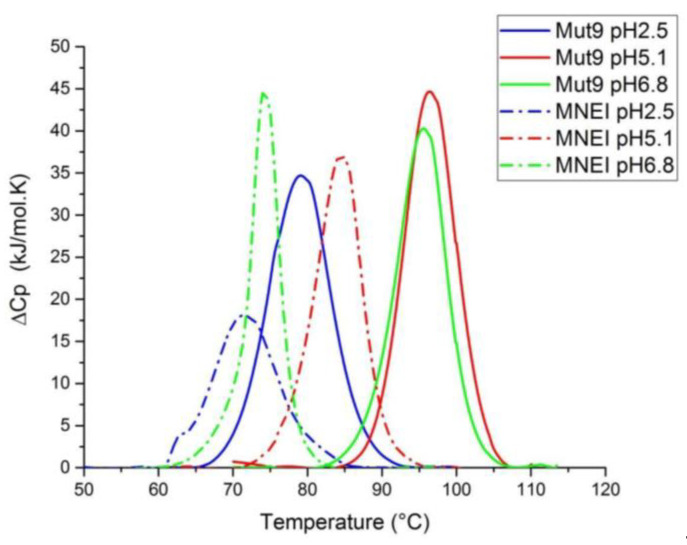
DCS thermograms of 1 mg/mL Mut9 and MNEI solution in 0.02 M sodium phosphate buffer recorded at different pH values.

The melting temperatures of Mut9 at the explored pHs were first evaluated by CD thermal denaturation experiments. The melting temperatures were found to be near or over the instrumental maximum limit temperature (95 °C) in all conditions except at pH 2.5. Therefore, Differential Scanning Calorimetry (DSC) experiments were carried out to assess and compare the T_m_ of Mut9 and MNEI. Figure 4 shows the DSC profiles obtained, and the corresponding thermodynamic parameters are collected in Table 2. Both MNEI and Mut9 thermal stability increased going from pH 2.5 to pH 6.8, with the T_m_ and the ΔH reaching a maximum at pH 5.1, indicating that both the parent protein and the new mutant favor slightly acidic pH over neutral and strongly acidic environments.

We also evaluated the reversibility of the thermal denaturation process by performing a reheating run of the samples after cooling down. The unfolding of Mut9 showed a very good reversibility at the acidic pHs, 2.5 and 5.1, whereas, at neutral pH, the denaturation process appeared irreversible. On the other hand, the denaturation of MNEI was reversible only at pH 2.5.

**Figure 5:**
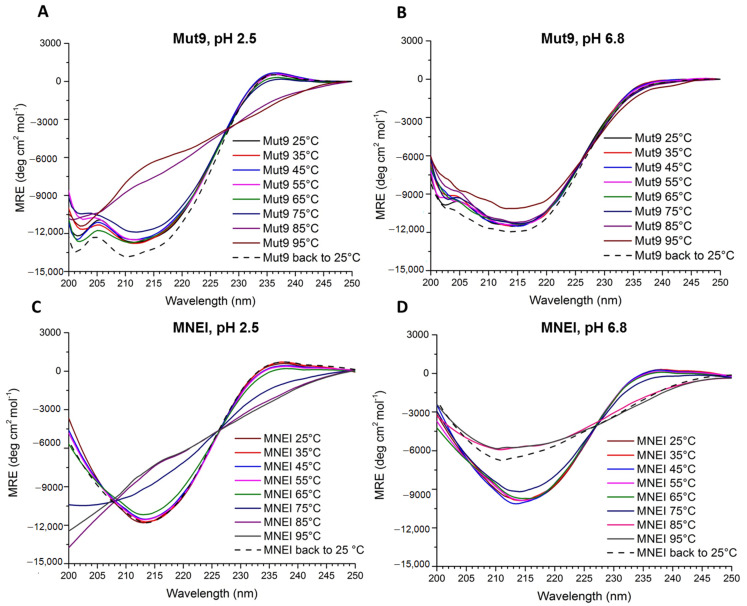
CD spectra of 0.2 mg/mL solutions of Mut9 and MNEI in 0.02 M sodium phosphate buffer. The presented data are: (**A**) Mut9 at pH 2.5, (**B**) Mut9 at pH 6.8, (**C**) MNEI at pH 2.5, and (**D**) MNEI at pH 6.8. The spectra were taken from 25 °C with 10 °C increasing interval to 95 °C and cooling back to 25 °C.

The stability and reversibility of the unfolding of Mut9 and MNEI were also evaluated by recording a series of CD spectra at acidic and neutral pH at increasing temperatures. At pH 2.5, the spectra of Mut9 remained unchanged until near the protein’s T_m_ (75 °C), whereas, at 85 °C, the line-shape of the spectra dramatically changed due to unfolding (Figure 5A). After cooling down the sample from 95 °C to 25 °C, the initial line-shape and intensity of the spectrum were fully recovered, indicating a reversible folding process. Regarding MNEI, the secondary structure at acidic pH was totally preserved until 65 °C, after which the protein started to unfold. Similarly to Mut9, MNEI refolded after cooling down the protein from 95 °C to 25 °C (Figure 5C). These results are in a good agreement with those from DSC. At pH 6.8, Mut9 presented a spectacular stability, and the spectrum of the folded protein was maintained at up to 95 °C, although, at this temperature, we observed a slight reduction of the spectral intensity (Figure 5B). Also at this pH, the spectra recorded before and after heating were superimposable, indicating the reversibility of Mut9 unfolding. On the other hand, MNEI was much less stable than Mut9: its unfolding process started above 75 °C (Figure 5D) and, at this pH, the protein did not refold after cooling down from 95 °C to 25 °C.

**Figure 6:**
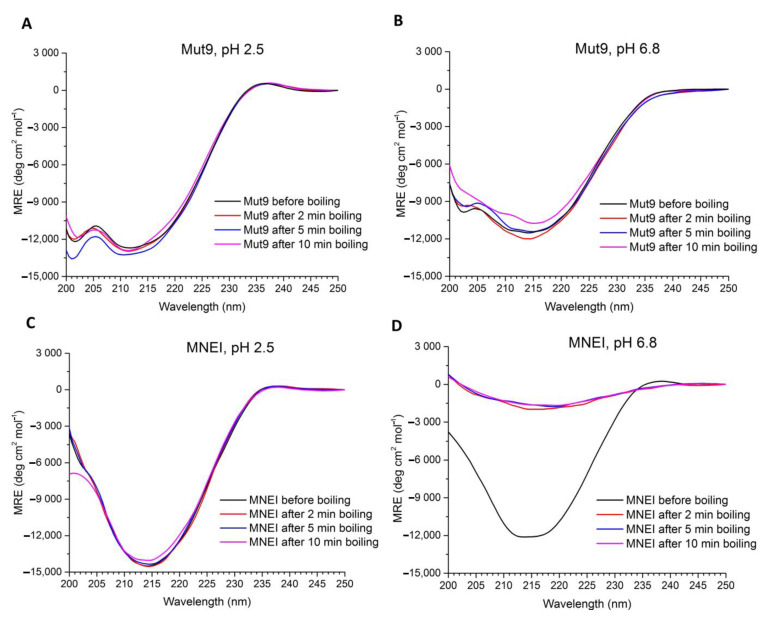
CD curves of Mut9 and MNEI with a concentration of 0.2 mg/mL in 0.020 M sodium phosphate buffer. The presented spectra are (**A**) Mut9 at pH 2.5, (**B**) Mut9 at pH 6.8, (**C**) MNEI at pH 2.5, and (**D**) MNEI at pH 6.8. This experiment performed under boiling conditions for 2, 5, and 10 min as reported in the inset of each spectrum. All the spectra were taken at 25 °C.

To gain further insight into the thermal and chemical stability of Mut9 in more severe conditions, samples of Mut9 and MNEI were dissolved at a concentration of 0.2 mg/mL at acidic or neutral pH and boiled for different times, cooled down at room temperature, and analyzed by CD spectroscopy (Figure 6). In fact, the CD spectra of both proteins, Mut9 and MNEI, at pH 2.5 remained practically unchanged upon boiling up to 10 min (Figure 6A,C). However, the difference in thermal stability was significant at pH 6.8 (Figure 6B,D). Indeed, the CD spectrum of MNEI at pH 6.8 was completely lost after only 2 min of boiling, very likely due to the high aggregation propensity of MNEI at neutral pH [50,53], whereas the secondary structure of Mut9 remained completely folded even after 10 min of boiling (Figure 6B). In addition, to understand how these differences in stability reflected the proteins functionality, we assessed the sweetness intensity of Mut9 and MNEI in the same experimental conditions and a protein concentration of 20 mg/L, i.e., a concentration lower than that used for the calorimetry and CD studies reported above, but closer to that of a potential drink. Consistently with CD data, both proteins at acidic pH preserved their sweetness upon 10 min of boiling, whereas at neutral pH, the sweetness of MNEI was completely lost after only 2 min of boiling; Mut9, instead, retained its sweetness intensity even after 10 min boiling.

### 3.5. Shelf-Life Assessment

**Figure 7:**
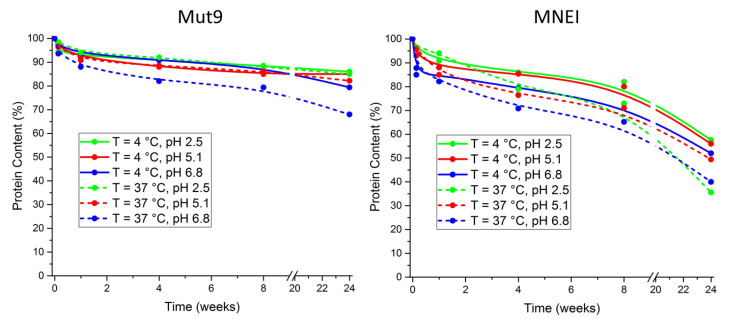
Shelf-life data presenting the protein content (measured by 280 nm UV absorbance) of Mut9 and MNEI as a function of incubation time at 4 °C and 37 °C upon 6 months incubation at different physicochemical conditions. Initial protein concentration was set to 5 mg/mL for all samples.

Long-term stability of Mut9 and MNEI was assessed by subjecting the proteins to prolonged incubation (up to six months) under various physicochemical conditions. We determined protein loss in the samples using UV spectroscopy. The proteins were incubated either at 4 °C, in order to simulate fridge storage temperature, or at 37 °C, to obtain an accelerated shelf-life assessment. Samples at different pH values (2.5, 5.1, and 6.8) and protein concentrations (0.5 and 5.0 mg/mL) were examined to evaluate the chemical stability and crowding effect of the proteins upon long-term incubations. Due to the negligible influence observed of the protein concentration on the stability and oligomerization tendency, only data at 5.0 mg/mL are presented. The results of these experiments are summarized in Figure 7. Even in this case, Mut9 displayed higher stability in all the conditions tested (Figure 7). In fact, samples of Mut9 incubated at 4 °C lost 10% of their initial protein content after the first 8 weeks, at all examined pHs; for the following 16 weeks, the protein under strong or mild acidic conditions remained totally stable, whereas, at pH 6.8 an additional 5% loss could be observed. In contrast, MNEI incubated at the same temperature lost approximately 45% (average of all conditions) of its initial amount after 6 months of incubation (Figure 7), showing the lowest stability upon 6 months incubation at pH 6.8. On the other hand, in the accelerated shelf-life assessment, Mut9 behavior paralleled that observed at 4 °C, while MNEI lost over 60% of its content upon 6 months incubation (average of all conditions).

## 4. Discussion

Sugar reduction in foods and beverages is an important objective worldwide, due to the diffusion of diabetes and obesity not only in developed countries, but also in emerging and less developed ones. In many food and beverage products, sugar has been replaced by non-nutritive sweeteners. However, these substances have recently been linked to severe health consequences [26]. Lately, natural sweeteners have attracted much attention from customers and food manufacturers. Among natural sweeteners, sweet proteins have great potential to replace sugar and other artificial sweeteners, but they also suffer from many limitations, due to poor availability and low thermal and chemical stability, which makes them unsuitable for certain preparations [11,42]. Monellin is one of the six sweet and sweet taste-modifying proteins found to date [2]. It is among the sweetest proteins known, being three orders of magnitudes sweeter than sucrose on a weight basis [42]. To overcome monellin lability, single chain variants were engineered, among which is MNEI, which presents a melting temperature 20 °C higher than the natural protein and comparable sweetness [40,41]. Single-chain monellin derivatives have additional advantages, such as easy, cheap and scalable production, absence of insulin release, high sweetness intensity, and low environmental impact [45,54,55]. Nonetheless, they still present drawbacks, particularly limited stability and high aggregations propensity at neutral pH. To overcome these limits, we reviewed existing literature data and designed a mutant, named Mut9, with improved properties in terms of sweetness, thermal stability and pH tolerance. To improve the sweetness, we introduced an extra positive charge at position 65, because we had already reported that the sole substitution Y65R decreased the sweetness threshold of MNEI from 1.645 to 0.665 mg/L [52]. Then, since this mutation is associated with a lower thermal stability at both pH 2.5 and 6.8 by an average of 7 °C compared to MNEI [50], we introduced other mutations, i.e., E23A [48] and S76Y [43], to enhance the stability of MNEI. The mutation S76Y was reported to improve the thermal stability by 10 °C with respect to MNEI [43]. Additionally, the substitution of E23 is known to increase the pH stability range of the protein, due to the peculiar position of this residue, located at the C-terminal region of the helix and buried inside a small hydrophobic cavity [48,51]. Finally, to further increase the pH stability, we replaced the only cysteine of the protein, i.e., C41, by alanine, thus removing the possibility of protein dimerization via an inter-chain disulfide, which could be facilitated by exposure to neutral and alkaline pH [48]. Hence, Mut9 contained four point mutations compared to the parent protein MNEI: E23A, C41A, Y65R and S76Y. We characterized Mut9 and MNEI using sensory and biophysical techniques, confirming the additivity of the features associated with the mutation introduced. In fact, according to the Y65R substitution, Mut9 appeared roughly twice as sweet as MNEI [52]. At the same time, the CD curves of Mut9 (Figure 2) were similar to those of the parent protein and were characterized by a high β-sheet content, indicating that the protein’s fold was unaffected by the mutations [56]. The stabilizing mutations introduced—E23A, C41A and S76Y—conferred extreme stability in a wide range of pH, and particularly at neutral pH, where the original protein is prone to denaturation and aggregation, whereas we observed a gain in stability of 21 °C (Figure 4). In terms of resistance to thermal degradation, DSC experiments demonstrated that both the T_m_ and the values of ΔH calculated for Mut9 were always higher than those of MNEI, at all values of pH explored. The greatest difference was observed at pH 6.8, where Mut9 presented a T_m_ over 20 °C higher than that of the parent protein. However, we observed a slight disagreement between CD and DSC data on the reversibility of the unfolding of Mut9 at neutral pH, which could be explained by chemical modification of the protein (e.g.*,* deamidation and other side reactions) promoted by the very high temperature reached (110 °C) to complete the unfolding process in the calorimetric study. The high stability gain in the case of Mut9 can be explained by the effect of the point mutations E23A and C41A that introduce apolar side chains into two distinct hydrophobic pockets that, instead, host ionizable/polar residues in the parent protein MNEI. This procedure is known to stabilize the native state of proteins, preventing unfolding, and, in the case of MNEI, was associated with a significant gain in chemical stability [48,51,57,58]. Another stabilizing point mutation was S76Y, which is believed to add more van der Waals interactions established between the tyrosine and its adjacent residues, leading to higher thermal stability [43].

When subjected to prolonged (up to 10 min) boiling, Mut9 was consistently more stable than MNEI, and preserved its sweetness longer even at neutral pH, where MNEI quickly precipitates, losing its sweetness after as little as 2 min boiling. These positive features were accompanied by additional functional gains, which emerged from the shelf-life study, where again Mut9 appeared much more resistant in solution than MNEI. Finally, the data obtained from the shelf-life study emphasized the excellent stability of Mut9 upon 6 months incubation in a wide range of physicochemical conditions. Mut9, in fact, remained mostly soluble, and MNEI lost more than half of its initial contents. Most likely, the loss of MNEI is due to aggregation and precipitation events, as suggested by the abundant white precipitate observed in the incubation vials. All these results strongly indicate that the mutations introduced in Mut9 result in a much more efficient construct, characterized by decreased aggregation propensity, wider pH tolerance, higher thermal stability and stronger sweetness, which could in principle be more compliant with industrial preparations and processes, thus being suited to replace sugar in food and beverages.

## 5. Patents

Patent application N. 102021000003698 is pending.

## Figures and Tables

**Table 1:** Secondary structure estimations for Mut9 at different pHs by spectral deconvolution. Errors on secondary structure content values are within ±2% [46].

	pH 2.5	pH 5.1	pH 6.8
α-helix	19.8	13.7	17.3
β-sheet (antiparallel)	42.2	38.3	38.8
β-sheet (parallel)	1.8	0	0
Turn	4.0	8.3	4.7
Random coil	32.2	39.7	39.2

**Table 2:** Thermodynamic parameters extracted from the DSC measurements. Errors on enthalpy and transition temperature are within ±5% and ±0.2 °C, respectively.

Properties	Mut9 pH 2.5	MNEI pH 2.5	Mut9 pH 5.1	MNEI pH 5.1	Mut9 pH 6.8	MNEI pH 6.8
Tm (°C)	78.0	71.4	96.2	84.4	95.8	74.2
ΔH_cal_ (kJ/mol)	327	241	384	253	336	247

## Data Availability

The data presented in this study are available in the article.
